# Reproducibility in Psychological Science: When Do Psychological Phenomena Exist?

**DOI:** 10.3389/fpsyg.2017.00879

**Published:** 2017-06-02

**Authors:** Seppo E. Iso-Ahola

**Affiliations:** Department of Kinesiology, School of Public Health, University of Maryland, College ParkMD, United States

**Keywords:** reproducibility, replication, meta-analysis, psychological phenomena, ego depletion

## Abstract

Scientific evidence has recently been used to assert that certain psychological phenomena do not exist. Such claims, however, cannot be made because (1) scientific method itself is seriously limited (i.e., it can never prove a negative); (2) non-existence of phenomena would require a complete absence of both logical (theoretical) and empirical support; even if empirical support is weak, logical and theoretical support can be strong; (3) statistical data are only one piece of evidence and cannot be used to reduce psychological phenomena to statistical phenomena; and (4) psychological phenomena vary across time, situations and persons. The human mind is unreproducible from one situation to another. Psychological phenomena are not particles that can decisively be tested and discovered. Therefore, a declaration that a phenomenon is not real is not only theoretically and empirically unjustified but runs counter to the propositional and provisional nature of scientific knowledge. There are only “temporary winners” and no “final truths” in scientific knowledge. Psychology is a science of subtleties in human affect, cognition and behavior. Its phenomena fluctuate with conditions and may sometimes be difficult to detect and reproduce empirically. When strictly applied, reproducibility is an overstated and even questionable concept in psychological science. Furthermore, statistical measures (e.g., effect size) are poor indicators of the theoretical importance and relevance of phenomena (cf. “deliberate practice” vs. “talent” in expert performance), not to mention whether phenomena are real or unreal. To better understand psychological phenomena, their theoretical and empirical properties should be examined via multiple parameters and criteria. Ten such parameters are suggested.

## Introduction

“The ego depletion effect” has recently been declared virtually dead or “indistinguishable from zero” (Carter et al., 2015), as has the actor-observer asymmetry in attributions for behavior (Malle, 2006). Is the demise of cognitive dissonance around the corner? What about anchoring effect, loss aversion, social comparison, confirmation bias, stereotype threat, self-affirmation, intrinsic motivation, psychological reactance, and countless other psychological phenomena? Are people about to stop “choking” as well?

It is curious that since its birth in 1998 (Baumeister et al., 1998), ego depletion was doing well until 2010 when one meta-analysis (Hagger et al., 2010) deemed the phenomenon not only alive but strong (Effect Size, ES = 0.62). Then, 6 years later, ego depletion stopped breathing thanks to another meta-analysis (Hagger et al., 2016). What happened in those 6 years? Is this what psychological phenomena are all about —they appear briefly and then vanish into black holes, or do we have a problem with methods and statistics used to determine phenomena’s existence? Although the purpose of this paper is not to analyze the existence/non-existence of ego depletion *per se*, but psychological phenomena’s existence and reproducibility in general, it should be noted that ego depletion’s demise is far from over. For example, Cunningham and Baumeister’s (2016) and Inzlicht et al.’s (2016) analyses revealed deep conceptual, methodological and statistical flaws not only in Carter et al.’s (2015) meta-analysis but in the practice of meta-analysis itself. These exchanges of criticism, however, are useful not only for clarifying the merits and limits of meta-analysis, but more importantly, for examination of the issue regarding the existence of psychological phenomena in general. When can we claim that a psychological phenomenon exists? Is it possible to declare that something does not exist? What are the criteria for concluding that something exists?

While a phenomenon and an effect can be closely related, they are not necessarily the same. That is, not all effects are phenomena. Sometimes the two, however, may be, and have been, used interchangeably. Any variable can have a significant effect on a criterion variable, but it would not automatically constitute a phenomenon. Although gender as a demographic or attribute variable could affect many psychological variables or have mediating effects, such influences in themselves would not constitute phenomena. However, gender bias is a psychological process that goes beyond simple gender effects on dependent variables and could therefore be labeled a phenomenon. Similarly, “choking” is a psychological phenomenon but a gender difference in the tendency to choke would not constitute a phenomenon, although it would clarify the phenomenon. More broadly, a phenomenon is a fundamental psychological process that has theoretically deduced antecedents and consequences and thereby helps explain human cognitions, feelings and behaviors. Whether ego depletion is an effect or a phenomenon is debatable. In this paper, for the sake of language, the two are used interchangeably.

## Non-Existence of Phenomena

When theoretical or empirical light has been shone on a psychological phenomenon (e.g., cognitive dissonance), is it possible to subsequently retract the phenomenon or worse, declare it dead? Following Zajonc’s (1965) influential paper, “social facilitation” became a popular topic of experimental research in the 1960s, 1970s, and 1980s, but little research is conducted on it today. Yet, the phenomenon is as important today as it was 50 years ago to explain the effects of others’ presence on learning and performance. Although Zajonc’s original hypothesis had to be refined with the cumulating evidence, the phenomenon itself has not been discarded or declared non-existent.

### Psychology as a Science of Subtleties in Cognition, Affect, and Behavior

There are two fundamental reasons why the retraction of known psychological phenomena is not possible. One has to do with the nature of psychological phenomena and the other with scientific method itself. First, psychology is a science of subtleties in cognition, affect and behavior. Its phenomena reside in and arise from the human mind, whether conscious or non-conscious, and as a result, are not static, but instead, dynamic and changing, varying with internal and external conditions. For example, thoughts and feelings often interact, one influencing another and both affecting behavior. Complicating the matters further, attendant thoughts are simple and complex at different times, and in some situations, both simple and complex at the same time. All of this means that there are no static phenomenon particles, unlike the Higgs Boson particle in physics. There is no ego depletion particle that, after exhaustive empirical work, can be said to exist or not to exist. Thus, in the absence of absolute constants regarding psychological phenomena in general, a target phenomenon is never zero, even if it is shown to be weak under some conditions.

Psychological phenomena exist to a varying degree, with variation occurring between and within them. Some of them are stronger and more consistent in their appearance and influence across time, situations and groups than others. It would be a mistake, however, to conclude that the former are more important than the latter, because they all are part of the psychological landscape helping explain the human condition and behavior. Human performers, for example, have to be able to deal with many psychological processes and phenomena to succeed. Of these, pressure is constant whereas “ironic errors” of mental control (Wegner, 1995) may be less so. However, if the latter occurs, its effects can be devastating for performers. Thus, some phenomena may appear less consistently across situations and yet be equally important as substantive phenomena. In short, inter-phenomenon differences have little relevance regarding the non-existence of a given phenomenon.

There is also variation within every phenomenon. For example, an experience of cognitive dissonance might be different for a non-exerciser after seeing a jogger on a day when he/she had earlier read about the importance of exercise for human health. On the next day, however, such environmental cues might elicit no dissonance. Would this mean that dissonance does not exist as a phenomenon because it failed to create dissonance consistently across all situations? Of course, it does not. Intra-phenomenon variation, small or large, would have no bearing on whether or not a phenomenon exists, but should invite empirical research to demonstrate the conditions under which a phenomenon is more likely to exert itself.

By their nature, psychological phenomena are subtle, elusive, and often brief in time. This is fundamentally due to conscious and non-conscious processing of cognitions and emotions. Thoughts and feelings vary not only between individuals but within a person, from one situation to another. Over time, cognitions and emotions are more variable than stable (Hudson et al., 2017). People’s thinking varies from simple to complex, from what might be called simple *main-effect thinking* to complex *interaction-effect thinking.* As a result, the threshold for emergence of phenomena can vary substantially. The same phenomenon may readily exert its influence in one situation but not at all in slightly different circumstances.

Priming studies illustrate this characteristic of psychological phenomena. On one hand, behavioral reactions can easily be produced by subtle non-conscious cues at one time in one specific situation, such as speaking softly after being exposed to a library picture (for a review of research, see Custers and Aarts, 2010; Baumeister et al., 2011; Kahneman, 2011; Bargh, 2014a). On the other, unless the individual conditions (e.g., a way of thinking) and social contexts are identical in other circumstances, the same unconscious social influences may not materialize at all (Bargh, 2014a). This would not mean that priming effects are weak but instead, that they are sensitive to subtle changes in the social environment. In fact, a long line of experimental research has shown that various forms of priming influence not only participants’ reactions in laboratory tasks but people’s behaviors in everyday life (Bargh, 2014b; Molden, 2014). However, the subtle nature and changing influences of psychological phenomena create methodological difficulties for research and also increase the likelihood of unjustified conclusion about phenomena’s non-existence.

The subtle nature of psychological phenomena and their sensitivity to social influences means that a previously observed effect may not appear under the seemingly same laboratory conditions at later times. It has been demonstrated that even minor differences in the wording of what were intended to be the same instructions could produce meaningful differences in outcomes (e.g., Bradley and Mannell, 1984). Furthermore, some phenomena are more likely to surface in the real world than in artificial laboratory conditions. For example, in a typical laboratory experiment on ego depletion, participants are asked to perform a simple short term task (e.g., to do something with their left hand) and are subsequently made perform an unrelated task. This sequential task performance is naturally different than a real life situation where a person uses his/her self-control skills all day long and then comes home exhausted from such a psychologically demanding work. It is logical to expect that the ego depletion effect is much more likely to occur in the latter than the former types of situations. Mani et al.’s (2013) seminal work speaks to this point. These researchers showed that poverty impedes cognitive function because the poor have to continuously make trade-offs in their daily living to survive, leading to reduced cognitive capacity and resultant impulsive and unhealthy choices.

Yet, all the experiments included in the reported meta-analyses are based on the former. Short-term artificial laboratory tasks hardly give a fair chance for the ego depletion effect to materialize, and therefore bias research against the existence of the phenomenon. In fact, it is surprising that any experiment would have produced any statistically observable effects. Yet, countless *experiments* from 1998 to 2010 supported the phenomenon. They all cannot be dismissed as methodological flaws, statistical flukes, and selective reporting, especially because they were published in the best journals of social psychology. How is it possible that all the peer-reviewers of these experiments would have made a fatal mistake of recommending them for publication during a period of 12 years? Most importantly, however, nobody has provided a theoretically and logically rigorous rationale and justification why ego depletion as a phenomenon should and would not exist.

Ego depletion is a good example for another reason, namely, lack of theoretical foundations to guide empirical studies. Lest we forget, ego depletion is but one form of self-control failure. It postulates that using self-control resources in one task leads to reduced capacity for self-control in a subsequent task. While the hypothesis is theoretically and logically justified, how does it compare to other reasons (e.g., lack of self-regulation skills) for self-control failures? Where does ego depletion fit in a theory of self-control failure? It could be argued that since no such theory has been developed, researchers have lacked theoretical guidelines, and have therefore had to focus on testing this simple hypothesis, which may ultimately turn out to be relatively inconsequential in a broader picture of self-control failure.

This is not to say that ego depletion is unimportant but to suggest that the phenomenon itself might benefit more from studies that would examine ego depletion’s effect in a broader network of relationships describing self-control failures. For example, it would be useful to know in what types of activities resource depletion is likely to occur, how much time and effort it takes before resource depletion effects begin occurring, etc. In the absence of such theoretically deduced parameters, researchers are forced to run simple experiments in artificial conditions where participants push buttons on computer screens and researchers subsequently determine, on a statistical basis, whether or not the null model is true. It almost sounds comical.

In this research approach, empiricists are potentially reducing a rich and complex phenomenon to a simple and isolated laboratory effect, studying the influence of a single independent variable on a single dependent variable. This is common of social psychological research in general, especially experimental studies. Yet, the real world is multi-causal, with phenomena being influenced by many causal factors. As Meehl (1990, p. 123) stated in his “crud factor” principle, “everything is correlated with everything, more or less.” This, of course, means that the null hypothesis is never true. That “everything influences everything” also means that psychological phenomena do not represent stand-alone effects but rather, are related to other effects. This in turn means that a manipulation of the focal independent variable affects, either by enhancing or reducing, other causal independent variables even in randomized experiments. When a researcher manipulates use of self-control in an experiment, he/she may simultaneously and unwittingly be manipulating participants’ tendency to compensate in subsequent tasks for their earlier poor performance in the first task; this compensation would subvert the ego depletion effect. Such uncontrolled causal factors can produce negative results. Taken together, the nature of psychological phenomena as subtle and correlated effects on one hand and their confounded manipulation in laboratories on the other, make it impossible to ever declare them non-existent.

Finally, it should be noted that psychological phenomena and their influences also vary in the long term, often becoming more pronounced and enhanced with time. “Persons are continuously and irreversibly changing” (Smedslund, 2016, p. 186). They learn from and change with their experiences, becoming “enlightened” by them (Gergen, 1973). Consider “choking,” an important psychological phenomenon. Pressure is the root cause of it (Baumeister and Showers, 1986; Lewis and Linder, 1997), but why is it that successful performers do not often choke? It is, of course, because they have learned to deal with pressure with an increasing number and type of experiences, and perhaps through psychological counseling, and those who don’t learn get weeded out. Thus, successful performers have become “enlightened” about pressure and its effects, and have learned to deal with it. This, however, does not mean that choking disappears into the black hole of non-existent psychological phenomena. For another example, studies (e.g., Jones and Stuth, 1997) have established that learning and practicing skills to visualize leads to improved performance, making imagery effects stronger with time and experiences. Taken together, psychological phenomena are not static but changing both in the short and long run. But to freeze phenomena in time to restricted laboratory conditions undermines their potential influence, thereby biasing results in favor of the null hypothesis. Such a methodology leaves out the most essential part of human life, namely, the interaction between individuals (Smedslund, 2016, p. 192).

### Limits of Empirical Science

Second, due to its nature, scientific method is limited in what it can do. It can never prove a negative, that something (e.g., God) does not exist, for a simple fact that it is impossible to investigate all conditions, take all confounds into account, and measure anything with perfect reliability and validity. Until our search has exhausted all the places where they could live, we cannot rule out aliens’ existence. Thus, there can be no categorical statements that something does not exist. Even in attempting to prove a positive, science can only provide conditional evidence, meaning that scientific knowledge is provisional and propositional. Thus, theoretically and logically deduced phenomena will always exist to a varying degree, with their empirical strength depending on methods used and conditions tested, but can never be shown to be zero across all conditions. As McFall (1996, p. 11) noted, since “there is an infinite number of ideas and ways to test them, no idea ever achieves the status of ‘final truth.”’

For example, self-control failures will always occur somewhere, in some situations and time, and among some individuals. In a similar vein, people will always experience, albeit to varying degrees, a phenomenon Festinger (1957) called cognitive dissonance. The theory’s tenets (i.e., hypotheses) can empirically be explored and tested, but even if some relationships predicted by the theory are not verified empirically in certain time and space, it does not mean that cognitive dissonance as a psychological phenomenon has ceased to exist and stop influencing human thought and behavior.

Psychological phenomena exist as theoretical constructs, not unlike those in physics. In 1964, the Higgs Boson particle was theorized to exist and permeate the entire universe, but it was not until 2012 that its existence was unequivocally proven in Large Hadron Collider in Switzerland. The measurement of the particle was possible only after particle colliders, detectors and advanced computers were developed. Although the particle’s existence could not empirically be proven for about 50 years, the particle nevertheless existed as a theoretical construct. The same applies to psychological phenomena, with one crucial difference: there are no particle phenomena in psychology that can conclusively be shown to exist, or not to exist. Psychologists can only accumulate as reliable and valid data as possible and demonstrate phenomena’s “track record” (Lakatos, 1971). But a poor track record does not necessarily mean that a phenomenon is weak, much less that it does not exist, because of lack of rigorous logical and theoretical development or because of methodological and measurement problems.

Strictly speaking, the inability of science to provide empirical proof for a negative does not mean that a negative could not occur, however unlikely it would be. Nothing has violated Einstein’s law regarding the speed of light, but it is possible that something could go faster than light, if not in the “classical reality” but in quantum reality. In fact, recent advances in quantum mechanics have hinted about this possibility. If physicists had not believed that violations of the speed of light are possible, perhaps their efforts to connect Einstein’s gravity with the quantum theory of atoms and molecules would have been discouraged permanently. Even if we cannot prove that aliens do not exist it does not mean they don’t. True scientists do not rule out any plausible alternative hypotheses.

Herein, however, is the difference between physics and psychology. The laws of the universe (and physics) are fixed and permanent, whereas in psychology no such laws exist regarding human behavior due to the elusive (changing) human being. As noted, human cognition, affect and behavior are in flux, and the speed of fluctuation does not matter. It is the task of theorists to postulate the conditions that cause stability and variation in phenomena and empiricists’ task to test the veracity of these postulates. All of this makes psychologists’ task much harder than physicists.’ We wish we could chase an ego depletion particle and finally find it, and then declare it one of the constants of the universe.

Kuhn’s (1962) “paradigm shifts” may occur in psychological science, but such shifts will not terminate psychological phenomena. If we move to cognitive neuroscience explanations (paradigm shift), these explanations only add to a more complete understanding of underlying phenomena (neural in this case), not their replacement by new ones. Undoubtedly, the neural basis of cognitive dissonance will eventually be shown. As a science, psychology is different from “hard” sciences in that it consists of a constellation of diverging states in human affect, cognition and behavior. As pointed out above, they are not fixed points in time and space, contrary to those found in physics and chemistry. In other words, this constellation of fluctuating phenomena defines the essence of psychology as a science, and these phenomena have no limits in time and space. They are not like Moore’s Law (i.e., a number of transistors on a microchip doubles every 18 months) that will come to a grinding halt in 2020. Psychological phenomena will never come to a grinding halt as long as humans are social animals. Whether various phenomena can be conflated into a unified theory of humans as psychologically functioning beings is anyone’s guess.

### Reproducibility and Replication

Recent emphasis on reproducibility is ill-guided because it is based on the assumption that for psychological phenomena to be real, they have to be stable from one context to another. It is further assumed that if phenomena are not stable they are not predictable. These assumptions have led to an overvaluing of stability and undervaluing of variability in psychological phenomena. Yet, as noted, psychological phenomena are inherently variable, even in artificial laboratory conditions (e.g., Bradley and Mannell, 1984). However, learning about the sources of stability and variability in phenomena is interesting and informative in and of itself, and theoretically, both can be predicted and determined equally well. Thus, there are two general approaches to reproducibility: (1) predict and determine the conditions for increased or decreased stability and (2) predict and determine the conditions for increased or decreased variability. The second is not simply the flip side of the first. Computing a stability coefficient over situations is nothing more than a test-rest reliability and as such, a limited way to understand a phenomenon’s nature, influence and repeatability, regardless of some researchers’ statements to the contrary (Simons, 2014). It reveals nothing about the conditions that produce stability. Consider the half dozen factors that have been identified to induce pressure (Baumeister and Showers, 1986) and their effect on choking. Which of these factors, or their combination, produces stability and variability in the effects of pressure on choking? It would be as important to know, for both theoretical and practical reasons, the conditions that produce variability as the conditions that produce stability in the effects, and why.

A problem with emphasis on stability is that it easily results in misguided research strategies in which investigators focus on determining a phenomenon’s stability across situations, persons and time. If stability is found to be relatively weak statistically, the phenomenon’s existence is questioned. Recent meta-analyses on ego depletion are good examples of this research strategy. Using a statistical criterion (ES), researchers have sought to determine whether or not ego depletion effects are “real.” Instead of stability, researchers would do better if they focused on finding out what causes variability in ego depletion effects, not whether the phenomenon exists.

As argued above, psychological phenomena, by their nature, are not fully reproducible. Cognitive dissonance exists in all humans but its expression varies within and between individuals, from one context to another, and as a function of time. More generally, the human mind is not reproducible because of its simplicity-complexity. In their thinking (conscious or non-conscious), people are simple at one time and complex at another time, or both at the same time. They can be astonishingly simple or irreducibly complex at various times. How does a replication researcher know the mode of thinking in which his/her participants are vis-a’-vis the participants of the original study? For this reason alone, reproducibility in psychology is unattainable. Moreover, “for complex systems (humans), all empirical inferences are false… by their assumptions of replicability of conditions, independence of different causal factors, and transfer to different conditions of prior observations” (Bar-Yam, 2016). Therefore, phenomena’s existence should not be defined by any index of reproducibility of findings, not to mention an evaluation of psychology as a science (Open Science Collaboration, 2015).

But if complete replicability becomes the main goal of psychological science, the only methodological option is to strip experimental participants out of everything to such a level of cognitive simplicity that they all act like robots. Although it is doubtful this could be accomplished, of what use would such experiments be anyhow? The best they could do is to demonstrate an arbitrary and artificial “time-limited situational stability,” but no permanent laws or “eternally valid principles” of psychological processes (Smedslund, 2016, p. 187).

It has been suggested that reproducibility is also unattainable because of problems and variations in “methods,” “results,” and “inferences” regarding replications of the original findings (Goodman et al., 2016). Methods Reproducibility refers to replication of the procedures and measurements of the original study, Results Reproducibility to replication of corroborating results, and Inferential Reproducibility to different scientists drawing the same conclusion from the same results.

Are studies methodologically reproducible? As Anderson et al. (2016) noted, there is no such thing as an exact replication because replication studies are conducted in “different facilities, in different weather, with different experimenters, with different computers and displays, in different languages, at different points in history, and so on.” Moreover, there are no perfectly reliable and valid measurement instruments in psychology. A typical reliability of dependent measures is about 0.70, which means that a retest shares only 49% of variance in common with the original test. Therefore, retest scores (e.g., mean differences, ESs) cannot be expected to replicate the original ones, especially given that reliability sets an upper bound on validity. In short, Methodological and measurement Reproducibility is impossibility.

Similarly, in the absence of consensus for the criteria of what constitutes a successful replication of *results*, and due to random error (sampling and measurement error) in findings, Results Reproducibility is unattainable. For example, if replicated studies draw their samples from different populations and use different procedures, as they often do, such “infidelities” produce random error (Gilbert et al., 2016) and as a consequence, Reproducibility of Results is compromised. Finally, Inferential Reproducibility poses major problems for replicating the original results because researchers often draw different conclusions from the same results (Goodman et al., 2016). Taken together, although reproducibility can be improved in replication attempts by increasing the reliability of data and the sample size of the original study (Stanley and Spence, 2014), reproducibility is generally unattainable for methodological, results, and inferential reasons.

Zealotry of reproducibility has unfortunately reached the point where some researchers take a radical position that the original results mean nothing if not replicated in the new data (e.g., LeBel and Peters, 2011; Francis, 2012; Simons, 2014). They believe that “direct replications test the basic existence of phenomena” (LeBel et al., 2017). Yet, there is no such thing as the basic empirical existence that can decisively be flushed out by “direct” replications. If the ego depletion effect is tested in a stripped-down experimental setting using an artificial short-term task, such contrived replications can say little about the basic existence of the phenomenon, only something about its emergence in these very limited conditions.

It is further believed that if better and more accurate measurements do not replicate the original finding, the effect is false. For example, Doyen et al. (2012) substituted laser beams for stopwatches to measure participants’ walking speed in an attempt to replicate the original behavioral priming effect (Bargh et al., 1996). When laser beams did not replicate stopwatches, the effect was declared “a methodological artifact.” While more accurate measures are desirable, they cannot eliminate other important reasons for failures to replicate, such as changes in the human mind and instructions given to participants from one study to another. Again, Methods Reproducibility is impossibility (Anderson et al., 2016; Goodman et al., 2016). Bar-Yam (2016) noted that “given the exponentially large set of possible environmental conditions, the chance that any particular condition will recur more than once is vanishingly small… using a controlled environment in experiments itself limits the study as a method for understanding the behavior.” In short, a failure to replicate does not mean that the original study was flawed; the flaws could be in replication studies.

Although full and conclusive replications are not possible, they can still be informative (Earp and Trafimow, 2015), especially “constructive” or conceptual replications (Lykken, 1968), as they attempt to establish a phenomenon’s boundary conditions. “Direct” replications, however, are problematic because they attempt to repeat a particular finding to a precise degree, which, as noted, is not possible for conceptual, theoretical and methodological reasons. Furthermore, success of direct replications is solely determined on a statistical basis, thus reducing psychological phenomena to statistical ones (Iso-Ahola and Dotson, 2015). Recent preregistered multilab replications are examples of this approach to replication. For example, Hagger et al. (2016) failed to replicate the ego depletion effect but, as cautious scientists, concluded that “it may be premature to reject the ego depletion effect altogether based on these data alone.” Indeed, this finding does not rule out ego depletion’s existence in other settings using different tasks and different participants, and different methodologies. Preregistered multilab replications, of course, are exercises in futility to the extent they are looking to find, in a critical test, whether or not an ego deletion particle exists. Furthermore, in efforts to eliminate tens of threats to internal validity (Shadish et al., 2002), they narrow replications of phenomena to a specific time and space, using a specific type of task in a particular manner. This “temporal situational stability” (Smedslund) is not only artificial and arbitrary, but undermines the discovery of the very essence of psychological phenomena; that is, how psychological processes shape human affective, cognitive and behavioral functioning under a variety of conditions.

Nevertheless, even failed and flawed replications can sometimes be useful and informative. Consider a long line of research on the contribution of “deliberate practice” vs. “talent” to expert performance. Based on this work, Ericsson and his associates (e.g., Ericsson and Ward, 2007) have claimed that deliberate practice explains about 45–50% the total variance in various domains of human performance. Consistent with this, one study (Meinz and Hambrick, 2010) found that the working-memory related ability (“talent”) to sight-read (to play music with little or no preparation) explained only 7.4% and deliberate practice 45.1% of the performance variance. But a recent replication through a meta-analysis (Macnamara et al., 2014) found the percentage for deliberate practice’s contribution to vary from 26% for games to less than 1% for “professions.” A closer inspection of their data, however, reveals that the analyses included middle school students, freshmen from college psychology classes, and nursing school students; mediocre performers such as middle-aged runners; and insurance sales agents and soccer officials as “professionals.” In contrast, Ericsson and his associates have studied expert and elite performers. Aside from its flaws, this replication meta-analysis (Macnamara et al., 2014) is nonetheless informative about the role of deliberate practice in non-expert performers. The phenomenon itself (deliberate practice in human performance) was replicated, even if not to the same magnitude as in other studies due to the methodological differences. In short, we can also learn from failed replications if they help us elaborate and expand our theories, provided that we do not use them to argue for the non-existence of phenomena.

### Falsifiability

Given that decisive replications are impossible, does it mean that psychological theories are unfalsifiable? In an absolute sense, it does because of theoretical and methodological problems discussed earlier, but also because of lack of absolute constants in psychology, such as the speed of light in physics. In psychological science, theories make predictions for relationships between variables, for conditions under which an effect is expected to be strong and weak, but nothing like the 99.9999999999+ accuracy offered by Einstein’s theory of general relativity for GPS satellites’ determination of one’s position on the earth. Moreover, tests and replications of psychological phenomena have a luxury of the third possibility, a suspended judgment. Empirical data can be accumulated without hard conclusions and theoretical foundations be improved in “continuous model expansion” (Gelman and Shalizi, 2013). Even the champion of the falsifiability principle of science (Popper, 1959) acknowledged that no conclusive disconfirmation of theories can be produced because of the unreliability of experimental results.

Nevertheless, if a theory predicts that a phenomenon is strong under certain conditions and weak under others, those conditions are clearly falsifiable and would therefore mean that the underlying theory is largely, though not completely, falsifiable. These conditions are “auxiliary assumptions” that connect a theory to empirically observable outcomes and thus make disconfirmation of theories possible (Earp and Trafimow, 2015). However, as these authors noted, absolute theory falsification and absolute theory verification are not possible because auxiliary assumptions alone can be false. And of course, all psychological theories are “imperfect in the sense of being incomplete… even the best theory is an approximation to the true state of affairs” (Meehl, 1990, p. 113–114). This suggests a difference between a falsification and an abandonment of a theory. Theories are abandoned only in Kuhnian paradigm shifts. The best that can be done is to accumulate as reliable and valid empirical data as the present methods allow while suspending hard judgments, but not denying a theory’s “poor public record” (Lakatos, 1971, p. 117).

However, it is not empirical data but theory that has generally made scientific progress possible. This is as true of physics as it is of psychology. Physics would not be where it is today without Einstein’s theories. Similarly, much of the progress achieved in psychology since its first experiment by Triplett (1898) can be attributed, not to a long list of empirical findings rejecting the null model, but to laudable theory- and model-building. Along the way, empirical data have complemented and contributed to the expansion of theoretical models, and theories have made data more useful. Bandura’s (1973) social learning theory and his experiments on Bobo Dolls are good examples of this. Given the severe limitations of empirical research, such as its ability to examine only a few of the large number of conditions of the human system’s response, theory-building and model construction become critical for scientific progress (e.g., Haig, 2017). This means that “an essential role of theory must be to identify which pieces of information are important” (Bar-Yam, 2016). More generally, it means that falsifiability and replication are of secondary importance to advancement of scientific fields.

### Statistical Appraisal of Theories and Phenomena

In psychology, the existence or non-existence of psychological phenomena is decided on the statistical basis, traditionally by means of NHST testing and the associated *p*-value (0.05). Recently, this decision has increasingly been reached using ES calculated from meta-analyses. After its invention (Glass, 1976, 1977) and subsequent expatiation (Hedges, 1981; Rosenthal, 1984; Hunter and Schmidt, 2004), meta-analysis has grown in stature. But it has only recently taken roots in psychology. This shift to meta-analytic persuasion has coincided with simultaneous calls for the complete abandonment of the sacrosanct *p*-value (e.g., Cumming, 2008, 2014). As a result, we now have a new magical weapon that is employed to declare when psychological phenomena exist and when they don’t. Researchers are running away from *p*-value to ES computed in meta-analysis, from one statistical tool to another. They are essentially embracing psychological phenomena as statistical phenomena, substituting statistics for psychology, while ignoring the importance of theoretical and applied relevance in basic and applied research, respectively. Perhaps researchers should stop to ponder Skinner’s succinct and brilliant argument against statistical significance testing altogether [paraphrased by Meehl (1990, p. 138)]: “If my work is replicable, the significance test is unnecessary; if my work is not replicable, the significance test is useless.”

If we nevertheless insist on statistical tests, a question is: Can theories and phenomena decisively be rejected or accepted by statistical criteria? The answer is no. Let’s first consider NHST as it has been the primary way of testing hypotheses and theories for decades even though many have called for its abandonment (e.g., Meehl, 1990). The problem with NHST starts with researchers’ unquestioned reliance on the *p*-value (*p*< 0.05) as a hard criterion for rejecting the null hypothesis. Unfortunately, *p*-value is often misinterpreted as the probability that the null hypothesis is true, which it is not. A significant difference observed between the two groups does not mean that the probability of a false positive is 5%, nor does it mean that the effect is likely to be true 95 times out of 100. More generally, a significant *p-*value (*p*< 0.05) does not indicate the probability that the null hypothesis is untrue given such an experimental finding but rather, the probability of this finding given that the null hypothesis is true (Trafimow, 2003). This distinction is important for attempts to correctly reject the null hypothesis or not to incorrectly accept it.

It is now well known that *p*-value fluctuates widely due to sampling variability (Cumming, 2008), thereby making it an unreliable indicator. Only at the 0.001 level, and possibly at 0.01, do *p*-values become reliable (Cumming), suggesting that 0.05 may be too liberal when used as a lone statistical criterion, not to mention that it can be obtained too easily in various ways (Simmons et al., 2011). However, it should be noted that not everything is *p’*s fault as it also reflects the influence of the underlying phenomenon and is therefore a measure of evidence. *P*-value is closely related to the data it summarizes but not the rate of error (alpha) applied to the test being performed (Haig, 2017).

The main problem with NHST is the null model itself and how it has been statistically tested. It is widely acknowledged that the null is always false (e.g., Lykken, 1968) because a target phenomenon is never zero in the real and complex world (“everything is somewhat correlated with everything,” Meehl). If so, a non-significant result cannot provide proof that the null hypothesis is true. The problem is compounded by methodological issues. That is, the null result could be due to a test that is not sensitive enough to pick up a population difference between the two groups being compared (Dienes, 2011). Furthermore, experimentalists can never control for all confounding factors and perform error-free experiments; every study has sampling error and measurement error. Thus, it is safe to say that a non-significant result likely leads to an incorrect acceptance (Type II error) of the null hypothesis (Dotson, 1980). But as Fisher (1925) himself advised, the null hypothesis cannot ever be proved. Accordingly, NHST does not provide evidence for the relative credibility of the null and alternative models (Kruschke, 2011).

What about refutation (e.g., a significant difference) of the null hypothesis and acceptance of an alternative hypothesis? Without specifying an alternative hypothesis, at best refutation would “corroborate the whole class of theories capable of generating a non-zero directional difference” (Meehl, p. 125). In other words, any *substantive* alternative hypothesis could be true. However, even if an alternative hypothesis is specified, it still is a statistical hypothesis, not a substantive or scientific hypothesis. Statistical tests performed under NHST are only tests of statistical hypotheses, not substantive hypotheses. Confirming evidence for a statistical alternative hypothesis does not constitute confirming support for a substantive hypothesis, although researchers frequently make this error. The two are not the same. This error-making tendency was evident in a reviewer’s question: “Why cannot we reject a substantive hypothesis by rejecting a statistical hypothesis, but by accepting a statistical hypothesis we accept the substantive alternative hypothesis”? Since the null and alternative hypotheses are statistical hypotheses, a substantive hypothesis cannot be accepted by rejecting the null or by accepting the alternative statistical hypothesis (Haig, 2017). The relationship between a statistical alternative and a substantive hypothesis is not automatic but instead, requires construction of the strong theoretical foundation for the correspondence. Showing theoretically which ideas are more likely to be true has clear implications for alternative statistical hypotheses to be tested.

Another major problem for verification and refutation of psychological effects comes from the fact that research cannot discover single causes that are both absolutely necessary and absolutely sufficient to explain human behavior. While deliberate practice is necessary but not sufficient for expert performance (Ackerman, 2014), psychologists are mostly trying to identify sufficient causes. For example, is the use of self-control resources in Task A a necessary and/or sufficient cause for reduced self-control capacity in Task B? It certainly is not necessary because there are other factors that can cause self-control failure (or reduction); whether the use of self-control resources will always result in subsequent self-control failures (a sufficient cause) is being debated. Although completely necessary and completely sufficient causes may separately be discovered in psychological research, as is the case for deliberate practice, we never discover both for a given phenomenon. This, in turn, means that the complete verification and refutation of psychological effects is unattainable, and no statistical test can change it.

Distinction has also been made between “a reason for thinking” and “a reason why” (Brennan, 2012). In other words, are we explaining a phenomenon “by citing a reason for thinking it is the case or by citing a reason why it is the case” (Brennan). For example, if there are many ways to measure self-control failure, then a change in the use of self-control resources will be a reason to think that a change in self-control failure has occurred, but not a reason why it has changed. Which of the two are we chasing in psychological research? Although it is difficult to make generalizations about necessary and sufficient causes, it is safe to conclude that unless we are able to find both necessary and sufficient causes, we can neither confirm nor deny phenomena’s existence. And no statistical test can change any of it. All of this further emphasizes that the most important work for advancement of science lies in theory-building, model generation, and “continuous model expansion” (e.g., Meehl, 1990; Gelman and Shalizi, 2013; Bar-Yam, 2016; Smedslund, 2016; Haig, 2017), not in the statistical testing of hypotheses.

All of the above is not to say that statistical testing should be abandoned completely. Rather, it calls for statistical pluralism, application of variety of statistical methods. In one approach, called the neo-Fisherian “error statistical philosophy” (Haig, 2017), tests of statistical significance replace NHST by complementing *p-*value with confidence intervals (CI) and ESs, but emphasizing the subservient role of statistical testing to theory construction. CI and ES are considered useful as they give more information about the data, with ES indicating a magnitude of the effect and CI showing a point and interval estimate of the population parameter. These techniques, however, are no panacea, for they have their own problems; for example, their values depend on sampling and measurement variability, *N*, statistical power, and the experimental design (within vs. between deign) (Lakens and Evers, 2014).

More recently, a Bayesian approach to statistical analysis has been advocated by many (e.g., Trafimow, 2003; Dienes, 2011; Kruschke, 2011; Earp and Trafimow, 2015), although cautions about it have also been expressed (e.g., Gelman and Shalizi, 2013; Haig, 2017). A “Bayesian evidence synthesis” approach (Scheibehenne et al., 2016) compares prior odds to posterior odds for a hypothesis given the data and distinguishes between (1) evidence for the absence of an effect and (2) the absence of evidence for an effect (Trafimow, 2003). It is worth keeping in mind that *p*-values, ESs, and Bayes factors “almost always agree about what hypothesis is better supported by the data,” though they may disagree about the strength of this support (Wetzels et al., 2011). Although the Bayesian approach is useful for testing alternative models, it is debatable whether the Bayesian analysis on its own provides more definite information about the truth of a hypothesis than other statistical methods. But it is an important additional tool available for statistical analyses, which in combination with other methods sheds further light on data. Comparison of various statistical methods, however, is beyond the scope of this paper.

#### Is Meta-Analysis the Solution?

Meta-analysis is promoted as the solution to the current reproducibility crisis. Some believe that “replication crisis perhaps exists only for those who do not view research through the lens of meta-analysis” (Stanley and Spence, 2014, p. 316). However, this method has many inherent flaws, such as the “apples and oranges” issue. Accordingly, studies representing different experimental designs, methodologies and measurement scales are thrown into the soup and the magic number (ES) is calculated. Voila! We have a phenomenon, or we don’t. If ES is different from zero, a phenomenon is declared to exist. And, if it does not, the phenomenon does not exist. Thus, we are back to the NHST testing and premature pronouncements that an effect is “indistinguishable from zero,” or that there is “very little evidence that the depletion effect is a real phenomenon” (e.g., Carter et al., 2015).

There are two problems with such sweeping conclusions. First, they were reached from a meta-analysis of studies conducted only in one type of experimental paradigm (i.e., the sequential-task paradigm). Yet, there is a multitude of other experimental and non-experimental paradigms where the depletion effect can and should be tested before anything could be concluded about the phenomenon’s non-existence. The sequential-task paradigm itself may be limited in its ability to generate evidence for the ego depletion effect (e.g., Lee et al., 2016), especially because self-control exhaustion is likely to be different in laboratory than real life tasks (Mani et al., 2013; Iso-Ahola, 2015).

Second, as Meehl (1990, p. 137) pointed out, meta-analysis is based on an erroneous assumption that the bigger the ES, the better. He argued that many theories, especially strong ones, make point or narrow-interval predictions of low “tolerance” for which the bigger-the-better ES would be inappropriate. The testing of a theory’s “intolerance,” however, is often obviated by methodological problems. If, for example, an experimental group is made to exercise only twice a week, a small sample size in such a study would be able to detect only large cardiovascular changes (e.g., VO_2_ max) when minimal changes would be expected with this light exercise regimen (Dotson, 1980). As a result, researchers would fail to test the theory’s low tolerance and be inclined to accept the false null that exercise does not improve cardiovascular health.

Meta-analysis advocates defend their method against the “apples and oranges” criticism by the test of homogeneity of ESs, that is, whether sample ESs are homogenous and come from the same population. If not, heterogeneous ESs simply reflect the presence of moderators, the influence of which can be shown. However, the validity of these moderators is in question because of methodological differences and errors observed in various studies used in meta-analyses; these errors cannot be wiped out by statistical tests within meta-analyses no matter how many and how varied they are, as demonstrated by Inzlicht et al. (2016). These authors performed 40,000 simulated meta-analyses using tests employed by Carter et al. (2015) and found them to be “unable to reliably discriminate between real and non-real effects, suggesting more broadly that meta-analyses, at least when the current crop of corrections is used, should themselves be treated with skepticism.” No matter how meta-analytic statistics are twisted, no generalized and definite conclusion about the effect’s non-existence can be made from meta-analyses. They are as limited as single experiments in this regard. Nor is the problem likely to be solved by “continuously cumulating meta-analysis” (Braver et al., 2014), because it still combines data from all the completed studies with methodological errors while computing meta-analytic indexes. Cumulated data by themselves do not wipe out methodological variations and errors of the original studies.

Meta-analysis, of course, is appealing because it allows for linear thinking. Since a meta-analysis is based on many studies it has to be better than one experiment in generating new knowledge, so goes the logic. “A single study often provides little information about the underlying true effect” (Stanley and Spence, 2014, p. 316). However, most meta-analyses are flawed as they do not correct for sampling error and measurement error. Schmidt (2010) reported that 79% of meta-analyses published in *Psychological Bulletin* were based on the fixed-effect model, which assumes that all differences between studies are due to sampling error, but not variation due to real differences in underlying phenomena across the studies. He further found that 90% of published meta-analyses did not adjust for measurement error. As a result, according to Schmidt, mean values and ESs are artificially reduced, and confidence intervals narrowed. Given such a wide-spread misuse of meta-analysis, how is one to have confidence in conclusions derived from these reports?

Moreover, how is a conclusion reached from a *single* (flawed) meta-analysis any better than the one reached from a *single* (unflawed) experiment? Why is a well-designed and conducted (double-blind, randomized trials, high statistical power) experiment not better than a meta-analysis based on 100 methodologically questionable studies? A patent response to this question is that 100 data points are better than a single data point, because 100 data points can be averaged and they can thereby get us closer to an underlying reality than one data point can (Stanley and Spence, 2014). However, averaging 100 data points from poor studies does not magically eliminate sampling and measurement errors present in those 100 studies. Meta-analyses are useful only to the extent that they are based upon studies with small sampling error, small measurement error, adequate sample size for high statistical power, and randomized experimental design. It should be noted that none of this is meta-analysis’ fault *per se*, but a serious problem arises when a heavy use and misuse of this statistical technique constitutes the basis for declaring the non-existence of psychological phenomena.

### Summary

Reproducibility in psychological science is unattainable for conceptual, theoretical, methodological, and statistical reasons. Psychological phenomena do not exist in social vacuum but vary situationally with subtle changes in conscious and non-conscious processing of cognition and affect. Human mind is dynamic and thus unreproducible from situation to situation. For this reason alone, it is not logically possible to declare that a psychological phenomenon does not exist. Psychological phenomena largely exist as theoretical constructs. Even if experimental conditions and instructions to experimental participants are standardized from one lab to another, a replication researcher cannot know the level of simplicity or complexity of cognitive functioning at which his/her participants are when performing a lab task vis-a’-vis participants of the original study.

Methodologically, there is no such thing as an exact replication because the conditions may vary substantially from one research setting to another by a variety of factors beyond researchers’ control. Moreover, researchers cannot control for all possible confounds; they cannot even think of all of them. In a complex interpersonal world, a manipulation of a focal independent variable, even in restricted lab conditions, simultaneously affects other causal factors. And of course, there are no perfectly, or even near-perfectly, reliable and valid measurement instruments in psychological science. All of this leaves every study with methodological and measurement errors and thus incapable of conclusive disproof or “strong inference” (Platt, 1964). Further, theoretical and methodological deficiencies cannot be saved by statistical analyses. There is no critical statistical test that can produce a numerical indicator that decisively declares the non-existence of the ego depletion particle (if there were one).

Despite these inherent difficulties of scientific inquiry, psychological science has made great progress not because of statistical significance testing but because of theory construction, hypothesis generation, and continuous model expansion. Although “statistical significance is the least important attribute of a good experiment” (Lykken, 1968), hypothesis testing (i.e., testing of alternative substantive hypotheses) is nevertheless essential for advancement of science. The overall goal is a rigorous theoretical and empirical examination of psychological phenomena from multiple perspectives, as suggested next.

## Parameters to Evaluate a Phenomenon’S Existence/Non-Existence

The ego-depletion controversy raises a broader question about psychological phenomena in general—when are they real versus unreal? The question, however, cannot be answered unless we are able to agree on what is real. For a phenomenon to be real, (1) Does it have to be theoretically relevant and important? (2) How stable does it have to be?; (3) How strong does its effect have to be and what percentage of variance does it have to explain?; (4) Does the dose-response effect have to be demonstrated?; (5) How frequently (every day, once a week, once a month, once a year?) do people have to experience it?; (6) How lasting and cumulative does its effect have to be?; (7) Do laypersons (and what percentage of them) have to be able to identify with or relate to it?; (8) How well does it have to compare to closely related phenomena, all of which are part of a larger underlying phenomenon?; (9) Does it have to be observed both in the “field” and in laboratories?; and (10) Does it have to be experienced consciously? In light of this multitude of parameters, it certainly is premature to argue for a phenomenon’s demise only on the basis of one statistical metric (e.g., ES).

All of these parameters should be taken into account in comprehensive reviews and analyses when the viability of various phenomena is examined. To this end, they all are critical, although the theoretical strength and relevance may be considered more important than the others. All the parameters, of course, cannot be addressed in a single study. However, when evidence obtained from all of them is pulled together, a picture about a phenomenon becomes much clearer; it also points out where more empirical work is needed. For one example, since most of the empirical work is done in laboratories (versus field settings) using artificial tasks on computer screens (Baumeister et al., 2007), what differences are there in emergence of the phenomenon in the two settings? For another, is the examined phenomenon a conscious vs. non-conscious experience, and to what extent? It has been theorized that self-control exhaustion resulting from a day’s work experiences leads to non-conscious choosing of the remote control and an inordinate amount of TV watching, which therefore in part explains a lack of exercise in the general population (Iso-Ahola, 2015). This, therefore, calls for studies to examine when ego depletion and other phenomena result in conscious versus non-conscious processing and resultant differences in affect, cognition, and behavior. In short, when all the suggested parameters are considered together, it becomes evident that evaluation of phenomena’s existence on the basis of one parameter (e.g., ES) alone is insufficient and inadequate.

### Theoretical Foundations

A good example of how a lack of theoretical underpinnings can derail and stifle scientific inquiry is research on psychological momentum, or “hot hand.” For about 30 years, a large group of statisticians and psychologists tried to empirically determine whether the phenomenon is real or just an illusion, with some studies supporting and others refuting it (e.g., Oskarsson et al., 2009). This is not surprising as the question cannot be conclusively answered, as noted throughout this paper, for empirical and statistical reasons. Rather than focusing on the yes-or-no question, greater scientific progress is made when time and effort is invested in theoretical elaboration and expansion of the phenomenon and subsequent empirical testing. The irony is that laypeople have known all along that psychological momentum is a real phenomenon as over 90% of them believe momentum to be a crucial determinant of different types of human performance, from sports to cleaning houses (Markman and Guenther, 2007; Iso-Ahola and Dotson, 2016).

Furthermore, psychological momentum is not an either-or phenomenon but consists of a variety of ubiquitous momentum-like experiences involving many theoretical properties from sensory processing to shifts in memory (Hubbard, 2015, 2017b). Theoretical analysis has even been extended to examination of musical inertia’s momentum-like effect as a general or unique mechanism of psychological and behavioral momentum (Hubbard, 2017a). Recent theory construction and model expansion (Iso-Ahola and Dotson, 2014, 2016, 2017) has also led to large-scale empirical investigations on elite performers.

First and foremost, psychological phenomena are theoretical phenomena. Empirical findings can only clarify and expand theoretical properties but never declare phenomena unreal or non-existent. Everyone agrees that depression is a real psychological phenomenon. This is because depression is theoretically well grounded and developed in terms of its depth and breadth. Countless studies and meta-analyses have been conducted on different aspects of depression, but not to determine whether or not it exists as a phenomenon. Similarly strong theoretical foundations have been established for many other phenomena (e.g., cognitive dissonance). A danger is that researchers with narrow statistical foci in their empirical studies tend to overlook or undermine the importance of theoretical relevance. Theoretical and conceptual replications (“constructive replications”) are different from experimental replications and can lead to great advances in science. All of this argues for the importance of evaluation of the theoretical strength and logical consistency of psychological phenomena. Thus, empirical studies are mainly evaluated for their theoretical relevance and importance, and less for their success or failure to exactly reproduce the original findings.

### Stability

Recent emphasis on reproducibility of psychological effects is driven by a false assumption that human behavior is stable (therefore reproducible) and that for psychological effects to be real, they have to be stable across time, situations and individuals. While some effects may be stable, others are inherently variable. Accordingly, depression varies with its severity, meaning that antecedents and consequences of mild depression are less reproducible than those of severe depression. Similarly, self-control may vary considerably during a day depending on situational demands. In fact, it is the characteristic of psychology as a science of subtleties in human affect, cognition and behavior that phenomena are variable, which then calls for an index of variability rather than stability. How variable are psychological phenomena? Aside from sampling errors, if Cumming’s (2014) data on the *p-*value’s fluctuations are an indication, they are quite variable. True variability, however, refers to variability in an underlying phenomenon, not variability due to sampling and measurement errors. Research should uncover factors that produce more variability and stability in some effects than others. Thus, it would be a mistake to determine a phenomenon’s existence on the basis of its stability or reproducibility across situations, time and individuals. More broadly, this calls for re-examination of the role of replication in psychological science.

### Strength of the Effect

#### Effect Size

Switch from *p-*value to ES has meant embracement of higher ES values as desirable because they supposedly reflect a greater magnitude of given effects. That is, the higher ES, the stronger is the effect. Although logically tenable, the inference poses difficult questions for claiming phenomena’s existence on the basis of ES (Meehl, 1990). This is especially true because no theoretically and logically derived psychological effect has a zero empirical effect. The null hypothesis is never true (Lykken, 1968).

To declare a phenomenon “viable,” how high does ES have to be or how low can it be? Can we regard any obtained ES number as an indication of a phenomenon’s existence as long as the associated confidence interval’s lower bound does not touch zero? If we can, we then have to accept that this determination would constitute a mathematical and statistical convention or even artifact, in principle no different than the *p* < 0.05 convention. In other words, a psychological phenomenon would be defined by mathematical reality, more specifically, by a simple statistic derived from subtraction of the control group’s mean from that of the experimental group divided by variability among participants. Variability (standard deviation) is, of course, directly affected by the sample size and the degree of controls within the experimental design.

Another consideration is that some effect-producing conditions demonstrate a ceiling effect. Accordingly, ego depletion would have a small effect in some but not in other tasks. For example, no matter how much self-control resources have been used in Task A, a person would likely exercise self-control not to make sexist and racist comments in Task B even if this task is baiting such comments. Other things being equal, the ceiling effect generally suppresses ES values and promotes the consequent bashing of the phenomenon in question. No such ceiling effects have been considered in previous empirical studies.

Finally, an important question is whether a phenomenon is examined as a dependent or independent variable. Do the conditions that precipitate the ego depletion effect show a stronger ES than an ES produced by the effect of ego depletion on other variables such as consumption of unhealthy food? In other words, ES is likely to vary depending on whether a psychological phenomenon is investigated as a presumed cause or a presumed effect or even the more complicated feedback effect. For example, does exercise have a greater effect (ES) on measures of depression (e.g., its remission and duration) than depression on indicators of exercise behavior, or measures with a wide range of “normal” (e.g., homeostasis) or measures including an exponential change where the change (effect) is greatly influenced by the starting measurement level and differs along the measurement change scale (Dotson, 1973)? A more complete understanding of psychological phenomena necessitates their examination as both an independent and dependent variables, as causes and effects.

#### Percentage of Variance Explained

Effect Size is essentially the same as percentage of variance explained by an independent variable. For decades, it has been the quantitative way to express the magnitude of an effect, and is often calculated as a standardized squared beta coefficient derived from regression analyses. Similarly, ES (e.g., eta square) shows the percentage of variance explained by the effect of an independent variable. In experimental research, variance explained is often small (under 10%) even though the *p-*value may be significant. A question, then, is: how much of the total variance should be explained by an effect for it to signify a meaningful influence? Many psychological phenomena are meaningful even if they explain only a small percentage of variance. For example, “talent” may explain only 7.4% of the performance variance, compared to 45% by deliberate practice, but this relatively small percentage is still critical for understanding exceptional performance (Ericsson and Ward, 2007; Meinz and Hambrick, 2010).

On a first glance, these percentages would seem to suggest that deliberate practice is the real phenomenon and talent is not. A problem with such comparisons, however, is that all along a scale of “ESs” or percentage-of-variance explained, the factorial structure defining a given “size” (i.e., score) is potentially different from other “sizes” and therefore, the interpretation of two different changes likely involves different “causes” or “effects” (Dotson, 1973). Echoing such interpretation problems, Ackerman (2014), in his informative analysis and review, further showed the fallacy of the percentage-of-variance comparison and called it “nonsense” and “silly” for conceptual and methodological reasons. For example, in studies supporting the deliberate practice explanation, data have artificially been restricted so that only talented performers have been included in analyses, thus not allowing for the influence of individual differences in talent to surface. This is tantamount, as Ackerman vividly illustrated, to calculating correlation between leggedness and swimming performance when including only two legged individuals at different levels of deliberate practice. Of course, the correlation will be zero regardless of 10,000+ hours of deliberate practice accumulated by performers with fewer than two legs. More generally, although practice is necessary for expert performance, it is not enough; nor is talent alone sufficient. Since both are critical determinants of expert performance, does it really matter if one explains somewhat more of the performance variance? Effects of both talent and deliberate practice are real phenomena.

### Dose-Response

In some areas of human behavior and performance, establishment of the dose-response effect can be useful. For example, regular light exercise (e.g., casual walking) produces benefits for physical health, but moderate exercise (brisk walking) more so, and vigorous exercise (jogging) even more. What about the dose-response in psychological effects? Since psychological effects can also vary on the linear continuum of more-is-better or more-is-worse (e.g., depression), determination of a phenomenon’s existence requires demonstration of dose-response effects. In fact, certain phenomena (e.g., fear of failure) may already exert their influence with a small amount, but the effect becomes more evident with greater amounts. For example, in competitive performance contexts, increased stress levels result in elevated cortisol levels in a linear fashion (e.g., Rohleder et al., 2007). In a different context, Festinger (1957) theorized that the magnitude of dissonance increases with the importance of dissonant elements. Thus, demonstration of the dose-response effect is helpful in better understanding psychological effects and making conclusions about the existence of various phenomena. It should be noted, however, that the dose-response effect does not have to be linear. It can also be quadratic (e.g., Baron and Kenny, 1986).

### Frequency

For a psychological phenomenon to be real, does it have to be experienced frequently? And if so, how frequently–daily, monthly, annually or once in lifetime? Csikszentmihalyi (1982), for example, reported that 30% of people experience “flow” daily. Is flow then a real psychological phenomenon for 30% of the population and no phenomenon for 70%? Many athletes have said that their best performance occurred when they were in “zone” (flow), but have not experienced it since then. Similarly, people relatively rarely may make “ironic errors” (Wegner, 1995) but when they do, the effects can be devastating on performance. Road rage as a psychological phenomenon of feelings of frustration and aggression may not happen often but when it does, effects can be destructive. Does the rarity of these phenomena make them less real?

Some phenomena are experienced daily, even if without cognitive awareness, such as causal attributions for one’s own and others’ behaviors. People who play tennis attribute the outcome to internal or external causes without conscious awareness. Are these frequently experienced phenomena more real? In some cases, frequency can be beneficial. For example, the more frequently psychological momentum is experienced *within* performance, the better the overall performance (Iso-Ahola and Dotson, 2014). What about frequently experienced feelings and moods, positive and negative, during a day? Are they therefore more real phenomena? In short, while frequency cannot alone be used to determine whether or not psychological phenomena are real, it can shed light on their nature and properties.

### Lasting and Cumulative Effects

Linear thinking would argue that the more lasting the effect, the more real is the phenomenon. There are, however, no theoretical or conceptual reasons for subscribing to this thinking because some effects are short-lived and others enduring. For example, the ego depletion effect, by definition, is temporary and transient as the self-control reserve is replenished after its exhaustion. Similarly, competition-induced stress dramatically increases the amount of cortisol in the blood stream before and during real-life contests, but equally dramatically disappears after competition (Rohleder et al., 2007), thus demonstrating the phenomenon’s strong but temporary effects. In contrast, effects of failure can be lasting and not easily eliminated. As a whole, all of this suggests that the lastingness and cumulativeness of effects cannot determine whether phenomena are real or unreal, although they can add to a better understanding of phenomena.

### Subjective Understanding

Do psychological phenomena have to be experienced and understood by most people before they can be considered real? Csikszentmihalyi (1982) reported that 13% of the sample could not identify with the characteristics of “flow,” whereas 87% indicated they can relate to the phenomenon. More generally, does 51% of the population have to endorse a phenomenon before it can be considered real, or are such percentages entirely irrelevant for determining a phenomenon’s existence?

As research on psychological momentum has demonstrated, subjectivity cannot be ignored. While scientists have continued to debate the phenomenon’s existence or non-existence, laypeople’s perceptions indicate that the phenomenon is real for them. Whether these perceptions are right or wrong from an objective scientific perspective does not matter because people act on their perceptions. If experimental evidence argues that the ego depletion effect is not real but non-experimental studies indicate that most people can “relate to” the effects of self-control exhaustion, which effect (experimental data or laypersons’ perceptions) is then real? Since psychological phenomena will always involve subjective perceptions, they cannot be ignored when the existence or non-existence of phenomena is discussed.

Relatedly, the above raises questions about scientists’ objectivity on one hand and their blind reliance on certain methods and statistical techniques in deciding what is real or unreal on the other. The concern is not psychologists’ problem alone. In the 1970s and 1980s, one group of astrophysicists in Texas found the rate by which the universe expands (Hubble constant) to be 100, while another group of scientists in California arrived at 50. For about 20 years, the two groups became so set in their views of how to measure distances to galaxies and the speed of receding galaxies that they could not accept an alternative to their own position. The research community was largely influenced by the reputation of these scientists, so much so that attempts to find a consensus figure for the constant were hindered (Begley, 1997). In the end, the orbiting Hubble Space Telescope settled the score: the constant is 75.

### A Phenomenon vs. Other Phenomena

Is a phenomenon’s comparability with other phenomena important in deciding on its existence and meaningfulness? Further, does it matter how well it relates to a larger phenomenon of which it is a part? Ego depletion is a part of a broader phenomenon called self-regulation failure (Baumeister and Heatherton, 1996). There are many factors that contribute to failures in self-control; one of them is ego depletion. Lack of self-regulatory skills (e.g., goal setting and monitoring) is another factor that has been shown to result in self-control failures. Similarly, motivational and attentional deficits lead to self-control failures (Inzlicht and Schmeichel, 2012). How does ego depletion stack up with these other factors? Assuming for a moment that it is less important in this regard, does this then diminish ego depletion’s value as a psychological phenomenon? On the other hand, if it is a larger contributor to self-control failures than most of the other factors, would ego depletion then become a more viable psychological phenomenon? More generally, is a phenomenon’s existence judged on its own merits or on a relative basis?

What about psychological phenomena versus non-psychological phenomena? Marsh and Perry (2005) reported that “previous best performance” explained over 80% of world-class swimmers’ performance variance in an international competition, whereas self-concept explained only about 10%; this 10%, however, was over and beyond the contribution of “previous best performance.” Although it numerically pales in comparison, self-concept still explained a meaningful and “significant” amount of variance. Notwithstanding the statistical significance, the 10% has real-world consequences. As the difference between winners and losers is often a matter of milliseconds among elite athletes, who of the top-level performers would not embrace an additional 10% increase in their performance resulting from enhanced self-concept? In other words, regardless of its small relative contribution to the total variance explained, self-concept is a real phenomenon in elite performance.

A recent experiment (Iso-Ahola et al., 2016) demonstrated the power of a psychological phenomenon relative to a non-psychological construct. In 2016, United States Golf Association banned golfers from “anchoring” their putter to their body (e.g., stomach), which would restrict free movement of hands and arms and thus potentially remove the influence of “nerves” from performance. Results indicated that anchoring improved performance only under high pressure, thereby showing that anchored putting gave a competitive advantage, not for a technical but psychological reason, in skilled performance. This empirical testing strengthened pressure as a theoretically important psychological phenomenon and expanded its theoretical properties. Such comparisons of psychological phenomena to non-psychological phenomena can be useful in better understanding the nature and strength of psychological phenomena.

### Laboratory vs. “Field” Effects

In general, since most psychological studies are done using the experimental method in laboratories, psychological phenomena could be dubbed as laboratory phenomena. Would they become more real if observed in real world? Baumeister, et al. (2007) expressed many researchers’ concern when they wondered what finger-press responses on computer keyboards have to do with real behaviors. Yet, almost all of the research on ego depletion has been done in laboratory settings using the dual-task protocol such that consumption of self-control resources in Task A (e.g., using one’s left hand) subsequently leads to reduced self-control capacity in Task B (e.g., eating chocolates). Carter et al.’s (2015) meta-analysis was only based on experiments testing the ego depletion effect in laboratories. Yet, there are field studies on the phenomenon. Hofmann et al. (2012), for example, employed the experience sampling method to collect data from students during a day and found that those who used their self-control resources more frequently and more recently were less able to control their desires later during a day. Iso-Ahola (2015) suggested that one of the best ways to test the ego depletion effect would be to measure consumption of self-control resources at a day’s work activity and then determine its effect on engagement in subsequent leisure activities such exercising and TV watching, with the suggested hypothesis that self-control exhaustion at work makes people less able to resist temptations and therefore more likely to participate non-demanding leisure activities (e.g., TV watching) and less likely to engage in demanding but healthy leisure activities (e.g., exercise). This is consistent with evidence that when self-control resources are depleted, people are less likely to perform non-habitual behaviors but continue to perform existing habits (Vohs et al., 2005).

There is no question that much more could be learned about psychological phenomena if they were studied outside of artificial laboratory settings. Ideally, the first line of research would test an effect in laboratories but would then move to natural “fields” and real-life situations to better establish the phenomenon’s external validity. It remains to be seen whether most, if not all, psychological phenomena are more powerful in real world.

### Consciously vs. Non-consciously Experienced Phenomena

Do psychological effects have to be experienced consciously for them to become real phenomena? Evidence is clear that conscious thoughts cause changes in behaviors (Baumeister et al., 2011). At the same time, non-conscious processing reliably influences cognition and behavior (Bargh, 2014a). Furthermore, some phenomena are originally experienced consciously but are later, with repeated exposure to relevant stimuli, relegated to non-conscious processing. For example, seeing joggers in a neighborhood makes non-exercisers and occasional exercisers experience cognitive dissonance for not exercising themselves. However, with repeated exposure to this stimulus (a jogger), occasional exercisers solve the arisen dissonance problem through non-conscious rationalizations (Iso-Ahola, 2013). Extending this to the ego depletion effect, to what extent has a person’s performance in Task B been influenced by non-conscious vs. conscious processing? The same question, of course, applies to experimental investigations of other psychological phenomena. Empirical findings could vary considerably as a function of participants’ conscious vs. non-conscious processing of task performance, which would in turn affect reproducibility of the findings and whether a phenomenon is declared real or unreal.

## Conclusion

For several reasons, as argued above, there is no such thing as non-existence of psychological phenomena. They exist to varying degrees and fluctuate across time, situations and persons. Theoretically well-developed concepts and effects (e.g., cognitive dissonance) define phenomena that will always exist in some time, contexts, and persons. Thus, psychological phenomena exist largely as theoretical constructs. Moreover, scientific method is fundamentally limited because it cannot prove a negative, that something does not exist. Scientific knowledge is provisional and propositional, meaning that there is no “final truth,” only “temporary winners.” It is therefore the task of empirical research to investigate conditions that cause changes in phenomena’s variability and strength, not whether they are real or not. In Ackerman’s (2014) words, it is “silly” and “nonsense” to expend energy in attempts to prove a phenomenon’s non-existence and non-meaningfulness on the basis of percentage-of-variance explained in relative comparisons of relevant effects.

When strictly applied, reproducibility is not only overstated as a scientific principle but remains questionable altogether in psychological science. There are two main reasons for this. First, reproducibility would have to be demonstrated in terms of “methods,” “results,” and “inferences” (Goodman et al., 2016), but the task is impossible for any studies. Second, by their nature, psychological phenomena are not stable across situations, tasks and persons, therefore being inherently non-reproducible. People are complex and elusive social beings. Given that they are sensitive to the influence of internal and external factors, their feelings, thoughts and behaviors vary across conditions. For example, one’s ability to exercise self-control may fluctuate considerably during a day depending on situational demands. Notwithstanding sampling and measurement errors, it is therefore impossible to re-create the precise conditions and contexts that produced specific thoughts and behaviors in the first place. Thus, variability should be embraced as it reflects the true nature of humans as psychological persons.

This is not to say that psychological phenomena are flimsy if they are not replicated successfully but rather, that their richness is revealed in their variability. Since it is the nature of psychological phenomena to vary between and within individuals, it becomes the goal of empirical research to examine factors that cause changes in emergence of phenomena and how they explain this variability. It is not to say that psychological effects would not have some, or even considerable, degree of stability. But this degree of stability, as expressed by any statistical indicator of reproducibility, cannot be a determinant of whether or not a phenomenon is real.

To better understand psychological phenomena, they can and should be studied as a function of many parameters other than their statistical strength (ES) and percentage of variance explained. Psychological phenomena cannot be reduced to statistical phenomena that are declared real or unreal on the basis of a statistical number obtained in specific space and time (Iso-Ahola and Dotson, 2015). “Deliberate practice” may explain many times more of the total variance in expert performance than does “talent.” Yet, talent is critical for understanding and explicating expert performance. If the magnitude of the effect alone were used as the criterion, talent would be rejected as a real phenomenon.

Instead, psychological phenomena should be examined for both their subjective and objective reality. We can learn much about a given phenomenon’s objective emergence and characteristics from how it is experienced and perceived subjectively. This understanding is further enhanced when phenomena are also studied in real world and “field” settings, not just in artificial laboratory conditions. Finally, it should not be forgotten that psychological phenomena are products of conscious and non-conscious processing of human thought and affect, meaning that one process can confound the effects of the other. While such effects can be empirically examined and even separated, they pose insurmountable difficulties for anyone to claim that a phenomenon (e.g., ego depletion) does not exist.

## Author Contributions

The author confirms being the sole contributor of this work and approved it for publication.

## Conflict of Interest Statement

The author declares that the research was conducted in the absence of any commercial or financial relationships that could be construed as a potential conflict of interest.
